# Segmentation of 71 Anatomical Structures Necessary for the Evaluation of Guideline-Conforming Clinical Target Volumes in Head and Neck Cancers

**DOI:** 10.3390/cancers16020415

**Published:** 2024-01-18

**Authors:** Alexandra Walter, Philipp Hoegen-Saßmannshausen, Goran Stanic, Joao Pedro Rodrigues, Sebastian Adeberg, Oliver Jäkel, Martin Frank, Kristina Giske

**Affiliations:** 1 Department of Medical Physics in Radiation Oncology, German Cancer Research Center (DKFZ), Im Neuenheimer Feld 280, 69120 Heidelberg, Germany; goran.stanic@dkfz-heidelberg.de (G.S.); joao.diasrodrigues@dkfz-heidelberg.de (J.P.R.); o.jaekel@dkfz-heidelberg.de (O.J.); k.giske@dkfz-heidelberg.de (K.G.); 2 Heidelberg Institute of Radiation Oncology (HIRO), National Center for Radiation Research in Oncology (NCRO), 69120 Heidelberg, Germany; philipp.hoegen@med.uni-heidelberg.de; 3 Karlsruhe Institute of Technology (KIT), Scientific Computing Center, Zirkel 2, 76131 Karlsruhe, Germany; martin.frank@kit.edu; 4 Department of Radiation Oncology, Heidelberg University Hospital, 69120 Heidelberg, Germany; 5 Clinical Cooperation Unit Radiation Oncology, German Cancer Research Center (DKFZ), 69120 Heidelberg, Germany; 6 National Center for Tumor Diseases (NCT), NCT Heidelberg, 69120 Heidelberg, Germany; 7 Faculty of Physics and Astronomy, University of Heidelberg, 69120 Heidelberg, Germany; 8 Department of Radiotherapy and Radiation Oncology, Marburg University Hospital, 35043 Marburg, Germany; sebastian.adeberg@uk-gm.de; 9 Marburg Ion-Beam Therapy Center (MIT), 35043 Marburg, Germany; 10 Universitäres Centrum für Tumorerkrankungen (UCT), 35033 Marburg, Germany; 11 Heidelberg Ion-Beam Therapy Center (HIT), 69120 Heidelberg, Germany

**Keywords:** automatic segmentation, anatomical structures, multi-label segmentation, clinical target volume delineation, lymph-node-level segmentation, expert guidelines, head and neck cancer

## Abstract

**Simple Summary:**

In radiation therapy, accurately contouring the volume containing cancerous cells is crucial for effective tumor control. Experts defined this volume by its borders with respect to anatomical structures. This study assesses the feasibility and precision of a deep-learning method in extracting 71 necessary anatomical structures from provided computed tomography scans. For most of these structures, automatically generated outlines are presented for the first time. For other structures, our model improves upon previously reported results. We analyzed the constraints posed by errors in the automatically generated outlines and found none that are relevant to our goal of identifying the entire volume containing cancerous cells. Our research contributes additional and enhanced outlines of anatomical structures, advancing the scientific objective of facilitating the contouring of a human’s complete anatomy. Moreover, confirming the effectiveness of automatic contouring techniques signifies a step closer to achieving precise automated contouring of the cancerous volume.

**Abstract:**

The delineation of the clinical target volumes (CTVs) for radiation therapy is time-consuming, requires intensive training and shows high inter-observer variability. Supervised deep-learning methods depend heavily on consistent training data; thus, State-of-the-Art research focuses on making CTV labels more homogeneous and strictly bounding them to current standards. International consensus expert guidelines standardize CTV delineation by conditioning the extension of the clinical target volume on the surrounding anatomical structures. Training strategies that directly follow the construction rules given in the expert guidelines or the possibility of quantifying the conformance of manually drawn contours to the guidelines are still missing. Seventy-one anatomical structures that are relevant to CTV delineation in head- and neck-cancer patients, according to the expert guidelines, were segmented on 104 computed tomography scans, to assess the possibility of automating their segmentation by State-of-the-Art deep learning methods. All 71 anatomical structures were subdivided into three subsets of non-overlapping structures, and a 3D nnU-Net model with five-fold cross-validation was trained for each subset, to automatically segment the structures on planning computed tomography scans. We report the DICE, Hausdorff distance and surface DICE for 71 + 5 anatomical structures, for most of which no previous segmentation accuracies have been reported. For those structures for which prediction values have been reported, our segmentation accuracy matched or exceeded the reported values. The predictions from our models were always better than those predicted by the TotalSegmentator. The sDICE with 2 mm margin was larger than 80% for almost all the structures. Individual structures with decreased segmentation accuracy are analyzed and discussed with respect to their impact on the CTV delineation following the expert guidelines. No deviation is expected to affect the rule-based automation of the CTV delineation.

## 1. Introduction

In the medical domain, the localization and determination of a disease’s extension can be a major advantage for the treatment. Ever since imaging modalities became available for cancer therapy, the precise delineation of organs and target volumes has been of great interest. The manual generation of these contours is thereby often time-consuming, requires intensive prior training and often lacks consistency between observers, especially for target volumes [1,2]. Because of the importance of available contour annotations in the clinical routine, a lot of research has been conducted in this area. Widespread early approaches that were used to automate medical-image segmentation were atlas-based methods [3,4,5]. For this, reference images were first contoured to build the atlas. These atlas images were then registered onto the new image while the same deformation field was applied to the atlas’ contours, resulting in a segmentation of the new image. While this approach proved to be successful in terms of manual labor reduction [6,7], it showed drawbacks in regard to individual segmentation quality, when the image quality or the individual anatomy deviated from the atlas.

With the increase in deep-learning (DL) methods that are capable of accurate contouring, the automatization of segmentation (auto-segmentation) has been applied in more and more of the areas in which medical images are analyzed. The most popular network architecture for automatic medical-image segmentation is the U-Net, which was introduced by Ronneberger et al. [8]. The deployment of this architecture in a framework with self-configuring hyperparameters, the nnU-Net [9], increased the accuracy and accessibility of DL-based segmentation methods. With the nnU-Net, it is possible to train a State-of-the-Art deep-learning model for medical-image segmentation tasks on custom data-label pairs, eliminating the need to explore task-specific hyperparameter settings.

While, at first, DL methods were optimized to predict single volumes of interest, the importance of models for multi-organ segmentation has increased [10,11]. Recently, the TotalSegmentator Version 2 toolkit was released under the URL https://github.com/wasserth/TotalSegmentator (accessed on 31 October 2023). The TotalSegmentator is a ready-trained open-access toolkit for the auto-segmentation of 117 anatomical structures in the whole body, which is based on the nnU-Net framework [11].

Multi-label-segmentation models have been shown to be beneficial for the segmentation accuracy of individual organs and for the robustness of the DL methods when compared to single-label models [12]. Currently, most multi-organ-segmentation models are trained on sparse labels (i.e., most voxels of an image are not labeled), due to missing dense annotations in the available medical-image data sets. In aiming to increase segmentation accuracy, the dense segmentation of the human body is necessary, i.e., the segmentation of every anatomical structure and its substructures. Gare et al. [13] showed that for ultrasound images dense pixel labeling improves disease classification when compared to models trained on only sparsely labeled images.

DL-based auto-segmentation enhances different tasks that need medical-image segmentation. Enhancements can be in the form of improved standardization, time savings or refined precision. Relevant tasks can be found in the realm of radiology, surgery [14] and radiotherapy. It also facilitates research fields like biomechanical modeling [15] and generation of synthetic medical-image data sets [16], which, in turn, improve the results in clinical applications. Nevertheless, the main application of automatic medical-image-segmentation methods lies within cancer diagnosis and treatment planning [17]. In cancer therapy, common auto-segmentation tasks are the segmentation of organs at risk (OARs) [18,19], target volumes [20,21,22,23] and metastases [24]. For example, Nikolov et al. [19] trained a DL-based auto-segmentation model that delineates 21 OARs achieving expert-level performance in the head and neck area.

In the field of radiation therapy, the exact contouring of OARs as well as target volumes is of major importance for the treatment outcome. Only with the precise delineation of target volumes and OARs, optimal tumor control can be achieved while adjacent healthy tissues are preserved. This significance is particularly pronounced in the head and neck region, where anatomical structures exhibit close spatial proximity paired with high anatomical flexibility. Target volumes as well as OARs are delineated by experts on the planning CT scans. These volumes are the basis for the objective function in the optimization of the radiation treatment plan.

Different target volumes are defined in radiotherapy. Following [25], the gross target volume is the visible and palpable, most inner tumor extension. It is surrounded by the clinical target volume (CTV) which comprises tissue that is potentially infiltrated by microscopic tumor cells. The CTV can itself be subdivided into the primary CTV and the nodal CTV. The primary CTV is drawn as a margin of 0.5–1 cm around the gross target volume, while the nodal CTV follows the lymphatic pathways and includes all areas that are found to harbor microscopic tumor cells with a probability of 10% or more [26,27,28]. The outermost target volume is the planning target volume which surrounds the union of all former mentioned target volumes and compensates for beam parameter uncertainties, patient placement errors, organ fluctuations and other motion-induced variance [29].

The extension of the CTV is not visible with modern imaging techniques, since it comprises normal tissues infiltrated by microscopic tumor cells. The definition of its outline is rather based on recurrence studies and thus, empirically built clinical experience [30,31]. This makes the delineation of CTVs a difficult task for clinicians that need many years of training [32]. Its complexity is not only visible in the training needed to perform this task, but also in the time needed to produce acceptable delineations and in their resulting divergence. Given the same CT scan, the manual CTV delineations of different experts show a large inter- and intra-observer variability of up to 200% difference in volume [1].

The quality of manual labels heavily affects the training and thus, the prediction accuracy of supervised learning methods. The inconsistent manual delineations of CTVs have a negative impact on the auto-segmentation of target volumes [33,34]. For that, researchers in this field focus on curating consistent data sets by executing extensive peer-reviews on the process of manual contouring or incorporating contours of only a minimum number of clinical experts, or institutes [21,22,23]. For CTV delineation, the predicted labels are reported to still need intensive pre- and post-processing [35,36,37,38] and they are not easily adaptable to changes in segmentation standards or patient-individual requirements. All this is done, aiming for improved spatial conformance of the predicted contour with manual delineation, while knowing that manual delineations are not well standardized.

Not only the comparison to labels that are highly dependent on the expert that generated the label, but also recent studies on evaluation metrics raise critiques on the current state-of-the-art. Reinke et al. [39] point out that the measurements of pure spatial overlap (i.e., the DICE) do not necessarily quantify the actual quality of interest in medical image segmentation tasks. For the delineation of CTVs the quality of interest that should be measured is the conformance of the CTV delineation with the expert guidelines.

To overcome the variety in CTV delineation, the detailed clinical knowledge about the extension of the CTVs is collected in international consensus expert guidelines including head and neck treatments [27,28]. These expert guidelines provide a commonly accepted delineation scheme for the CTVs in a rule-based manner and thus, standardize their segmentation. As one example, Grégoire et al. [27] focus on the delineation of nodal CTV in the head and neck area. In these expert guidelines, the nodal CTV is subdivided into ten levels with some additional subdivisions. The extent of each single level is described by bordering anatomical structures. Thus, the expert guidelines convert the difficult problem of delineating the extent of cancerous infiltration which is not visible in CT scans, in a contouring task of anatomical structures. The selection of levels that should be irradiated is based on the location of the primary tumor.

In summary, the current status quo for automatic CTV delineation is to optimize a metric that measures spatial conformance with unreliable manual labels that impair the training of supervised learning methods. The inconsistency of the manual labels result from the diverse character of cancer growth and the missing contrast to surrounding tissues. The international consensus expert guidelines are based on the combination of anatomical boundaries for which more consistent segmentations are expected. Thus, we advocate the exploitation of written-down human knowledge-based expert guidelines as ground truth for the CTV delineation overcoming the dependence on inconsistent manual labels and solely focusing on commonly agreed standards.

Oriented towards the goal of evaluating guideline conformance of CTV delineations, in this study, the 71 most important anatomical structures mentioned in the expert guidelines have been chosen for an auto-segmentation task. For that, all 71 structures have been manually delineated, and used to train nnU-Net models for auto-segmentation. The predictions for 18 unseen data sets are evaluated against the manual labels as well as segmentations generated by the TotalSegmentator, and compared to previously reported segmentation results. So far, studies on the segmentation of anatomical structures have only published results on a small subset of the necessary 71 anatomical structures. The existent results are widely distributed over multiple unrelated publications.

In this study, 48 of our 71 anatomical structures are automatically segmented for the first time. For the remaining structures, our model provides improved or comparable segmentations. We evaluate the segmentation accuracy between different tissue types and reasons for why some structures are more difficult for an auto-segmentation task. Finally, the impact of the segmentation accuracy for the construction of CTV delineation according to the expert guidelines is discussed. Our results indicate that the automatic application of delineation rules given in the expert guidelines is feasible without any restraint.

## 2. Materials and Methods

### 2.1. Image Properties of the Data Set

**Figure 1:**
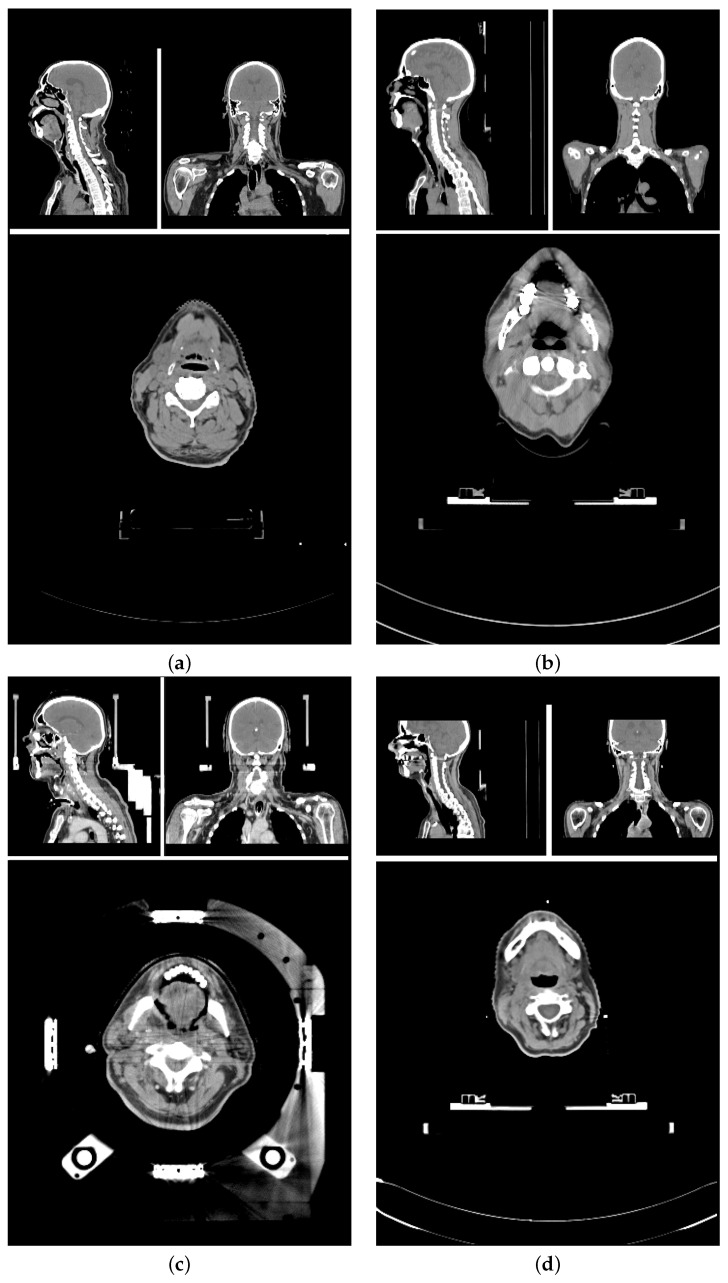
Screenshots of planning CT scans from exemplary patients of all four cohorts in sagittal, coronal and transversal view. (**a**) Open access HNC data set [40,41,42], (**b**–**d**) in-house HNC data sets. All cohorts differ in their scanning set-up using different treatment couches and immobilization devices. (**b**) Shows artifacts due to dental implants, and (**c**) shows artifacts due to the stereotactic frames and underwent tracheostomy.

The planning CT scans for this study were aggregated from four different study cohorts. Figure 1 shows an exemplary CT scan of each cohort. All patients received radiotherapy for head and neck cancer. For each patient, there was exactly one planning CT scan considered in this study. Each CT scan consists of 90 to 220 single slices (mean: 141 ± 24) of 512 × 512 voxels each. The voxel size ranged from 0.98 × 0.98 × 2 ${\mathrm{mm}}^{3}$ to 1.27 × 1.27 × 3 ${\mathrm{mm}}^{3}$ .

The training data set and test data set are mutually exclusive. The *training data set* (86 scans) included (a) 84 in-house HNC patients from three different cohorts (varying setup, positioning, devices, and protocols) [43,44], and (b) 2 open access HNC data sets [40,41,42]. The *test data set* (18 scans) is curated from the same three study cohorts (14, and 4 scans, respectively). The patient selection for the test data set was based on available meta-information to best represent the variety of the data cohorts. Factors for the selections were study cohort, location of the primary tumor, gender, presence of a tracheostoma, size of nCTV, estimated age and weight of the patient.

### 2.2. Label Selection and Generation of the Manual Labels

**Figure 2:**
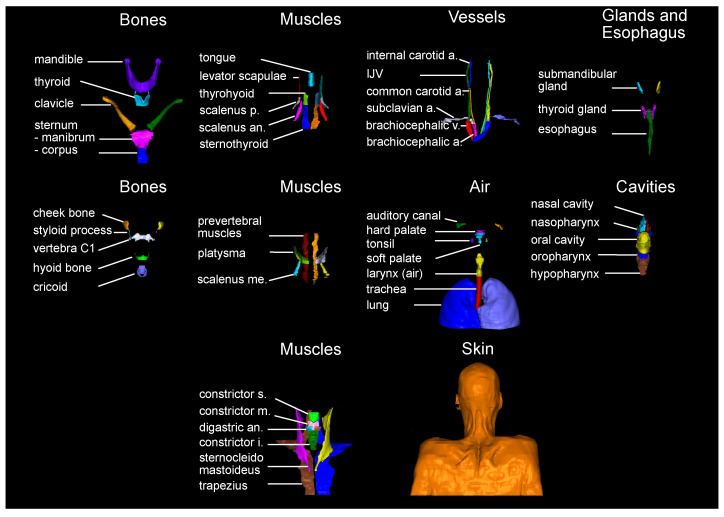
Visualization of all 71 anatomical structures manually delineated. Abbreviations: a. artery, an. anterior, i. inferior, m. middle, me. medius, p. posterior, s. superior, v. vein.

The 71 structures were chosen based on their number of occurrence in the Grégoire et al. [27] expert guidelines. The resulting set of anatomical structures is visualized in Figure 2. Manual labels of the 71 anatomical structures were generated for all 104 CT scans by six different trained observers on a Wacom Cintiq 24HD Display in RayStation 8B(R) SP1. The observers were following a standard operation procedure for the delineations that included (a) the unambiguous definition of the structures’ extent (e.g., mandible without teeth), (b) windowing, and (c) spatial restrictions based on other anatomical structures (mostly cranial and caudal). The whole standard operation procedure can be found in Appendix A.1. Each data set was at least once reviewed and if necessary adjusted by one of the other observers before it was accepted for the study.

For one patient data set, 41 selected structures were segmented a second time by one of the trained observers who was not involved in the initial segmentation or the review of this patient. Based on those two sets of contours, the inter-observer variability was approximately assessed.

Caused by the field of view of our CT scans, the esophagus, the sternum (corpus and manubrium), the lobes of the lung, the trachea, the trapezius muscles, the brachiocephalic veins, and the skin are never or not always completely present on our patient scans, but cut off on the caudal edge of the scan. The sternum corpus is sometimes not present at all. Further, in cases where the patients were post-operatively irradiated, or the extension of the primary tumor distorted surrounding anatomical structures, the respective missing anatomical structures were not segmented. In total, there were 30 anatomical structures missing. Fifteen of those structures cumulated in two test patients (#8, #7), and three other patients had at least two missing structures. Nine of the 18 test patients were not missing any structure and thus, had the full set of 71 anatomical structures manually segmented.

### 2.3. Network Training and Label Prediction

For the automatic segmentation, the nnU-Net framework Version 1 was chosen and trained with one adaption to the default parameters: mirroring was removed from the data augmentation to keep the left-right orientation of the patients consistent during training. The final training data set provided for the nnU-Net training was generated by mirroring all 86 training data sets. Left and right instances of anatomical structures were then swapped back for left-right consistency after mirroring.

Since in the nnU-Net Version 1, a network can only be trained for non-overlapping structures, the labels of all 71 anatomical structures were subdivided into three non-overlapping, disjoint subsets, containing (a) the labels for all bones, muscles, vessels, air-related structures, glands and the esophagus (#64), (b) the labels for all cavities (i.e., hypopharynx, left and right nasal cavity, nasopharynx, oral cavity, and oropharynx), and (c) the skin label. According to the author, nnU-Net Version 2 has no accuracy advantages over its Version 1 [45].

Following the nnU-Net’s five-fold cross-validation standard, for all three subsets there were five 3D full-resolution models trained with the trainer V2. Fold 1 and fold 2 were using 137 data sets for training and 35 data sets for validation, while fold 3–5 were using 138 data sets for training and 34 data sets for validation. Each fold was trained for 1000 epochs. The predictions were made for all 18 previously unseen test data sets in the nnU-Net’s default 5-heads manner. No postprocessing was applied.

All computations were executed using the nnU-Net Version 1.7.0 with Python Version 3.9.7, PyTorch 1.10.2 with CUDA Version 11.3.1. Training and predictions were executed on a computer with an AMD Ryzen™ 9 3900X Processor, 128 GB RAM, with an NVIDIA GeForce RTX 3090, and 24 GB VRAM.

For 16 of our anatomical structures, segmentations can also be retrieved by using the pre-trained TotalSegmentator toolkit. We employed the TotalSegmentator as Python library on our 18 test patients with default configurations. The predictions generated by the TotalSegmentator were run on a computer with an Intel^®^ Core™ i7 Processor, 64 GB RAM, with an NVIDIA GeForce RTX 2070, and 8 GB VRAM.

### 2.4. Evaluation of Predicted Labels

We assess the similarity and distance between two distinct labels of the same structure through three metrics: (a) their volumetric overlap, measured using the Sørensen–Dice coefficient (DICE) [46,47], (b) the distance between both contours, evaluated by the Hausdorff distance (HD) [48] and (c) the fraction of deviation larger than 2 mm, quantified using the surface DICE (sDICE) as defined in Nikolov et al. [19]. For the evaluation of the HD we chose the 95th percentile (HD (95)). Choosing a margin of 2 mm is based on the clinical practice in photon radiation therapy to intervene when deviations are in the order of 2 mm or larger. The sDICE (2 mm) is considered to indicate the correction effort needed for the predicted CTVs. This selection of metrics is consistent with the metrics reloaded framework [39] accessible under the URL https://metrics-reloaded.dkfz.de/ (accessed on 20 October 2023). Structures that are not present in the manual labels, in the predicted labels or both sets of labels are left out in the analyses. For the calculation of all metrics, the library surface-distance-based-measures Version 0.1 was used.

## 3. Results

### 3.1. Analysis Based on Volumetric Overlap

**Figure 3:**
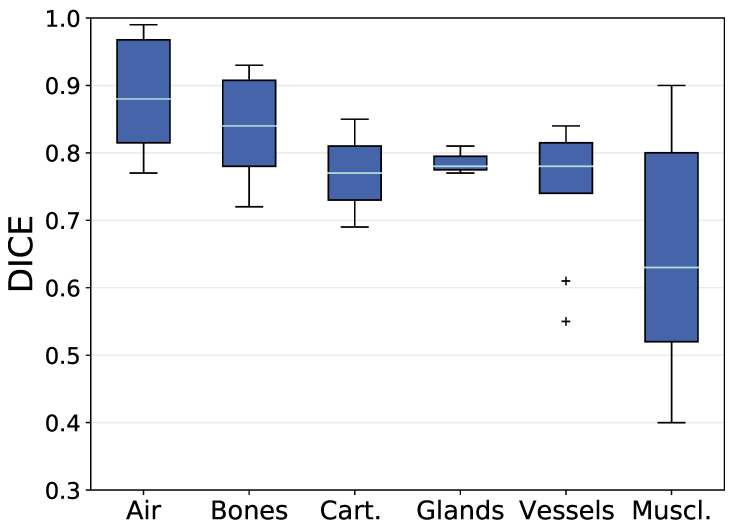
Mean DICE values between manual delineation and predicted label for each anatomical structure grouped by their tissue types. Means are calculated over all test patients for that the structure is present (maximum 18 test patients). Box plots show the median (cyan) and outliers (cross). Box (blue) reaching from the first quartile (Q1) to the third quartile (Q3), whiskers reaching to the 1.5 interquartile range. Quantities per group were: Air (6), Bones (11), Cartilages (2), Glands (3), Muscles (26), and Vessels (11).

An overview of the volumetric overlap between the manually segmented and the predicted anatomical structures is given in Figure 3. It shows the mean DICE (DICE_m_) value for each anatomical structure over all test patients grouped by their tissue types. The median and standard deviation of the DICE_m_ is 0.88 ± 0.09 for air-related structures, 0.84 ± 0.07 for bones, 0.77 ± 0.08 for cartilages, 0.78 ± 0.02 for glands, 0.78 ± 0.09 for vessels, and 0.63 ± 0.16 for muscles. Outliers are left and right internal carotid arteries. The box plot of all muscles is wide spread, while all other box plots show a centered median with symmetric and narrow distribution of DICE_m_ values around it. The analysis will focus on structures that are below the 25th percentile (Q1) in DICE_m_ within the group of muscles. This comprises all single parts of the constrictor muscle, the right digastric muscle, the left and right posterior scalene muscles, and the left thyrohyoid muscle.

A precise evaluation of the volumetric overlap between the manually segmented and the predicted anatomical structures is given in Table 1. It shows the DICE_m_ value for each anatomical structure over all test patients, as well as the inter-observer variability in DICE and previously reported DICE values for comparison. Some of the individually segmented 71 anatomical structures form a meaningful unit together, i.e., they are substructures of a coherent anatomical structure. Thus, Table 1 also contains (a) the *sternum (M., C.)*, a combination of the sternum manubrium and the sternum corpus, (b) the *constrictor muscles (s., m., i.)*, a combination of the inferior, the middle and the superior constrictor muscle, (c) the right and left *scalene muscles (an., me., p.)*, a combination of the right and left anterior, medius and posterior scalene muscle, respectively, and (d) the *pharynx (nasop., orop., hyp.)*, a combination of the nasopharynx, hypopharynx and oropharynx. With these combinations, Table 1 contains a total of 76 anatomical structures.

The inter-observer variability is approximated for 45 selected structures and their available combinations. Inter-observer values outside the 3 σ interval around the DICE_m_ are indicated by an asterisk (^*^). Although within the 3 σ interval, the inter-observer DICE is noticeably low for the left internal carotid artery, the left and right posterior scalene muscles, the left and right digastric muscles, and the tonsils.

Table 1 also shows previously reported DICE_m_ values. While for most structures, there is no DICE value found for comparison (48 of 76 structures), or only a single reference (17 of 76 structures), there are multiple comparisons for 11 anatomical structures. Detailed values for multiple comparisons are listed in Appendix A.2. Our prediction results are mostly within the 3 σ interval (single comparison) or within the given range (multiple comparisons). Lower DICE_m_ values than previously reported result from the internal carotid arteries, and the inferior, middle and superior constrictor muscle. For the former, left and right instances are jointly evaluated in Nikan et al. [49], Ke et al. [50], while for the latter, our results are comparable to Thomson et al. [51], Van Dijk et al. [52] when all substructures are combined. Higher DICE_m_ values than previously reported result from the levator scapulae muscles, and the prevertebral muscles, and the *sternum (M. C.)*, which is not completely present on our CT scans.

**Table 1:** List of all segmented anatomical structures (right (r), left (l)) and their combinations (e.g., *sternum (M., C.)*) sorted by tissue type. For each structure, the DICE (mean ± standard deviation) between the manual contours and our models’ predicted contours (pred.) is given, as well as the inter-observer variability in DICE (calculation based on a single patient data set). Asterisks (^*^) indicates inter-observer variability values outside the 3 σ interval given by the mean and standard deviation of the models’ comparison to the manual labels. The last column shows DICE previously reported results as mean ± standard deviation (single comparison) or the range of means (multiple comparisons). Superscript numbers indicate differences between the structure’s definition in the literature and the definition used in this paper. Explanations are found as footnote at the end of the table.

	Structure	Pred. vs. Manual	Inter-Observer	Literature
Air	Auditory Canal (l)	0.77 ± 0.09		0.83 ± 0.02 [50] ^2^
	Auditory Canal (r)	0.80 ± 0.10		0.83 ± 0.02 [50] ^2^
	Larynx (air)	0.86 ± 0.06		
	Lung (l)	0.99 ± 0.01		0.98 [53] ^1, 2^
	Lung (r)	0.99 ± 0.01		0.98 [53] ^1, 2^
	Trachea	0.90 ± 0.07		
Bones	Cheek Bone (l)	0.78 ± 0.04		
	Cheek Bone (r)	0.78 ± 0.06		
	Clavicle (l)	0.93 ± 0.02		
	Clavicle (r)	0.93 ± 0.01		
	Hyoid Bone	0.82 ± 0.07	0.76	
	Mandible	0.88 ± 0.06	0.78	[0.86–0.99] [52,54,55,56,57]
	*Sternum (M., C.)*	0.93 ± 0.04		0.83 [58] ^1^
	Sternum Corpus	0.82 ± 0.22		0.90 ± 0.03 [59] ^1^
	Sternum Manubrium	0.90 ± 0.06	0.88	
	Styloid Process (l)	0.72 ± 0.14		
	Styloid Process (r)	0.77 ± 0.08		
	Vertebra C1	0.86 ± 0.04	0.84	
Ca.	Cricoid Cartilage	0.69 ± 0.15	0.78	0.66 ± 0.12 [52]
	Thyroid Cartilage	0.85 ± 0.06	0.85	
Gland	Submandibular Gland (l)	0.77 ± 0.17		[0.70–0.97] [51,52,54,55]
	Submandibular Gland (r)	0.78 ± 0.13		[0.73–0.98] [51,52,54,55]
	Thyroid Gland	0.81 ± 0.13		0.83, 0.90 [52,57]
Vessels	Brachiocephalic Artery	0.84 ± 0.06	0.85	
	Brachiocephalic Vein (l)	0.82 ± 0.10	0.77	
	Brachiocephalic Vein (r)	0.82 ± 0.07	0.76	
	Common Carotid Artery (l)	0.81 ± 0.08	0.72	0.84 ± 0.04 [57] ^2^
	Common Carotid Artery (r)	0.78 ± 0.10	0.50	0.85 ± 0.03 [57] ^2^
	Internal Carotid Artery (l)	0.61 ± 0.15	0.25	0.81, 0.86 [49,50] ^3^
	Internal Carotid Artery (r)	0.55 ± 0.22	0.49	0.81, 0.86 [49,50] ^3^
	Internal Jugular Vein (l)	0.78 ± 0.13	0.45	
	Internal Jugular Vein (r)	0.75 ± 0.18	0.53	
	Subclavian Artery (l)	0.74 ± 0.09	0.54	
	Subclavian Artery (r)	0.74 ± 0.13	0.34 ^*^	
Muscles	*Constrictors (s., m., i.)*	0.56 ± 0.12	0.74	0.52, 0.68 [51,52]
	Inferior Constrictor	0.44 ± 0.16	0.54	[0.65–0.80] [55,60]
	Middle Constrictor	0.45 ± 0.18	0.66	[0.60–0.84] [55,60]
	Superior Constrictor	0.48 ± 0.19	0.42	[0.67–0.83] [55,60]
	Digastric (l)	0.52 ± 0.24	0.39	
	Digastric (r)	0.46 ± 0.28	0.33	
	Levator Scapulae (l)	0.87 ± 0.05		0.76 ± 0.01 [61]
	Levator Scapulae (r)	0.83 ± 0.07		0.76 ± 0.01 [61]
	Platysma (l)	0.59 ± 0.12		
	Platysma (r)	0.52 ± 0.16		
	Prevertebral (l)	0.74 ± 0.07	0.53 ^*^	0.70 ± 0.01 [61]
	Prevertebral (r)	0.76 ± 0.06	0.50 ^*^	0.71 ± 0.01 [61]
	*Scalene (an., me., p.) (l)*	0.74 ± 0.09	0.44 ^*^	
	*Scalene (an., me., p.) (r)*	0.71 ± 0.11	0.03 ^*^	
	Anterior Scalene (l)	0.82 ± 0.06	0.60 ^*^	
	Anterior Scalene (r)	0.80 ± 0.06	0.00 ^*^	
	Medius Scalene (l)	0.68 ± 0.10	0.14 ^*^	
	Medius Scalene (r)	0.66 ± 0.16	0.03 ^*^	
	Posterior Scalene (l)	0.40 ± 0.20	0.01	
	Posterior Scalene (r)	0.42 ± 0.28	0.00	
	Sternothyroid (l)	0.58 ± 0.08		
	Sternothyroid (r)	0.59 ± 0.09		
	Sternocleidomastoid (l)	0.84 ± 0.07	0.51 ^*^	0.73 ± 0.02 [61]
	Sternocleidomastoid (r)	0.81 ± 0.15	0.52	0.74 ± 0.02 [61]
	Thyrohyoid (l)	0.50 ± 0.17	0.48	
	Thyrohyoid (r)	0.56 ± 0.12	0.56	
	Trapezius (l)	0.90 ± 0.03	0.65 ^*^	0.41 ± 0.04 [61]
	Trapezius (r)	0.89 ± 0.04	0.72 ^*^	0.45 ± 0.04 [61]
	Tongue	0.63 ± 0.17		
	Esophagus	0.80 ± 0.10		[0.55–0.83] [52,55,57] ^4^
	Hard Palate	0.63 ± 0.13		
	Hypopharynx	0.64 ± 0.15	0.71	
	Nasal Cavity (l)	0.86 ± 0.03		
	Nasal Cavity (r)	0.86 ± 0.03		
	Nasopharynx	0.83 ± 0.09	0.74	
	Oral Cavity	0.85 ± 0.07		[0.85–0.93] [52,55,57]
	Oropharynx	0.84 ± 0.09	0.83	
	*Pharynx (nasop., orop., hyp.)*	0.82 ± 0.07	0.83	0.69 ± 0.06 [54]
	Skin	0.99 ± 0.00		
	Soft Palate	0.61 ± 0.19		
	Tonsil (l)	0.08 ± 0.13	0.12	
	Tonsil (r)	0.12 ± 0.15	0.15	

Differences between the structure’s definition in the literature and the definition in this paper: ^1^ The structures mentioned in Section 2.2 are not completely present on each patient scan within our data set, whereas the literature references are using scans containing those structures completely. ^2^ In the literature, internal, external and common carotid artery are jointly delineated. ^3^ In the literature, left and right instances are jointly evaluated. ^4^ In the literature, only the upper [55] and cervical esophagus is segmented [52].

### 3.2. Analysis Based on Distance-Based Metrics

**Figure 4:**
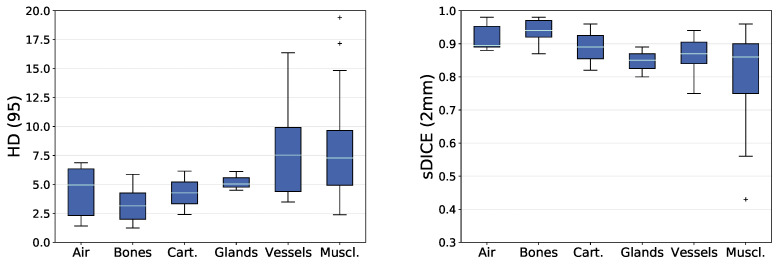
Mean HD and mean sDICE values between manual delineation and predicted label for each anatomical structure grouped by their tissue types. Means are calculated over all test patients for that the structure is present (maximum 18 test patients). Box plots show the median (cyan) and outliers (cross). Box (blue) reaching from the first quartile (Q1) to the third quartile (Q3), whiskers reaching to the 1.5 interquartile range. Quantities per group were: Air (6), Bones (11), Cartilages (2), Glands (3), Muscles (26), and Vessels (11).

An overview of the distance-based metrics between the manually segmented and the predicted anatomical structures is given in Figure 4. It shows the mean HD (95) (HD_m_) and the mean sDICE (2 mm) (sDICE_m_) for each anatomical structure grouped by their tissue type. The median and standard deviation of the HD_m_ is 4.96 ± 2.22 for air-related structures, 3.15 ± 1.51 for bones, 4.28 ± 1.88 for cartilages, 5.04 ± 0.67 for glands, 7.53 ± 4.13 for vessels, and 7.29 ± 4.23 for muscles. The median and standard deviation of the sDICE_m_ is 0.90 ± 0.04 for air-related structures, 0.94 ± 0.03 for bones, 0.89 ± 0.07 for cartilages, 0.85 ± 0.04 for glands, 0.87 ± 0.05 for vessels, and 0.86 ± 0.13 for muscles. Outliers in HD_m_ are the right platysma muscle and the right posterior scalene muscle. The outlier in sDICE_m_ is the tongue.

For the HD_m_, the analysis will focus on structures that are above the 75th percentile (Q3) within the group of vessels and the group of muscles. This comprises the right internal carotid artery, the left and the right subclavian artery, the right sternocleidomastoid muscle, the superior constrictor muscle, the left platysma muscle, and the left posterior scalene muscle. For the sDICE_m_, the analysis will focus on structures that are below the 25th percentile (Q1) within the group of vessels and the group of muscles. This comprises the left and the right internal carotid artery, the right subclavian artery, the middle and the superior constrictor muscle, the left and the right digastric muscle, and the left and the right posterior scalene muscle.

A precise evaluation of the distance-based metrics between the manually segmented and the predicted anatomical structures is given in Table 2. It shows the HD_m_ and the sDICE_m_ for all 71 segmented anatomical structures and the five combinations over all test patients, as well as the inter-observer variability in HD (95) and sDICE (2 mm). The inter-observer variability is calculated for the same subset as described for the DICE. Inter-observer values outside the 3 σ interval around the HD_m_ and sDICE_m_, respectively, are indicated by an asterisk (^*^). Although within the 3 σ interval, the inter-observer HD (95) is noticeably low for a variety of scalene muscles, and the tonsils. For the DICE and sDICE (2 mm), structures of low overlap are the same.

### 3.3. Completeness of Predicted Label Set

In the 18 test patients’ anatomies, a total of 30 anatomical structures are absent. Thirteen of these 30 structures were correctly identified as missing anatomical structures by the trained nnU-Net models (true negatives). The remaining 17 missing structures were erroneously contoured (false positives). Amongst these 17 structures, the sternothyroid muscle was contoured five times, the platysma muscle three times, and the posterior scalene muscle two times.

The analysis of anatomical structures that were present in the test patients’ anatomy, but not segmented by the trained nnU-Net models (false negatives), result in the model’s capability to predict all but two of the present structures (larynx (air), posterior scalene muscle (l)). The tonsils were excluded from this analysis, since they are generally difficult to segment as indicated by the inter-observer variability which is shown in Table 1 (DICE) and Table 2 (HD, sDICE). They were predicted correctly on both sides only in eleven of the 18 test patients. Even when predicted, the overlap between manual and predicted segmentations was small.

### 3.4. Analyzing Only Patients without Tracheostoma

In the training data set, approximately one third of the patients were scanned with a tracheostoma. In the test data set this ratio is one sixth, respectively. Although trained on several data sets with tracheostomy, test patients that have a tracheostoma show below-average values in several anatomical structures. Table 3 lists the 17 most deviating structures. For these structures, the DICE_m_, HD_m_ and sDICE_m_ is shown when only patients without tracheostomy are considered. The deviation of all metrics between this analysis and the analysis considering all patients is presented in brackets. All structures beside these 17 anatomical structures show low deviations between both analyses: the average deviation is 0.00 ± 0.07 in DICE_m_, and −0.01 ± 0.07 in sDICE_m_.

### 3.5. Comparison to TotalSegmentator

Applying the pre-trained TotalSegmentator framework (TS) to our data resulted in predictions of 16 common anatomical structures. Thereby, our label ‘Brachiocephalic Artery‘ corresponds to their ‘Brachiocephalic Trunk’. All 16 structures are listed in Table 4 which shows the DICE_m_ comparing the TS predictions with our manual segmentations. Differences between this comparison and the comparison of our predictions to the manual labels are favoring segmentations generated by our models (i.e., all values are negative). Below the Q1 of −0.10 for the difference in DICE_m_ is the trachea, the thyroid gland, and the left and right common carotid arteries.

Table 5 shows the same comparisons using the HD_m_ and the sDICE_m_. All predicted segmentations generated by our models show better results in HD_m_ (i.e., all diff. values are positive) and better or equal results in sDICE_m_ (i.e., all diff. values are negative or zero). Above the Q3 of 7.98 for the difference in HD_m_ is the trachea, the left and right common carotid arteries, and the right subclavian artery. Below the Q1 value of −0.09 for the difference in sDICE_m_ is the trachea, the thyroid gland, and the left and right common carotid arteries.

## 4. Discussion

When comparing the grouped DICE_m_ between tissue types, groups with good contrast on CT scans like air-related structures and bones show an increased accuracy when compared to other groups. Noticeably, the variation in DICE_m_ is the largest for the group of muscles. First, this group has the largest number of different anatomical instances. Further, the contrast of soft tissues on CT scans is not sufficient to identify most muscles completely. Finally, the group of muscles is also the most diverse group ranging from structures with an average volume of 550 voxels (digastric muscle) to 55,000 voxels (trapezius muscle).

### 4.1. Reasons for Impaired Prediction Accuracy

We have visually analyzed cases of impaired prediction accuracy for highlighted anatomical structures from before. Typical deviations occur at the transition between related structures (e.g., between the superior, the middle and the inferior constrictor muscles), or at the beginning and ending of elongated structures (e.g., the final cranial slice of the internal carotid artery). DICE values are sometimes low for thin structures although the sDICE (2 mm) is high. This is because small deviations of thin structures can lead to a large decrease in overlap and cause large changes in DICE, which does not tolerate any type of deviation. The sDICE (2 mm) instead allows deviations smaller than 2 mm. Non-systematic segmentation errors originate from largely deviating manual labels, which are cause by metal artifacts (e.g., for the tongue) or insufficient soft tissue contrast (e.g., for the platysma muscle). In the following section, reasons for impaired prediction accuracy are discussed for every prior identified anatomical structure, for that the automatic prediction resulted in a below Q1 (or above Q3) evaluation metric.

**Figure 5:**
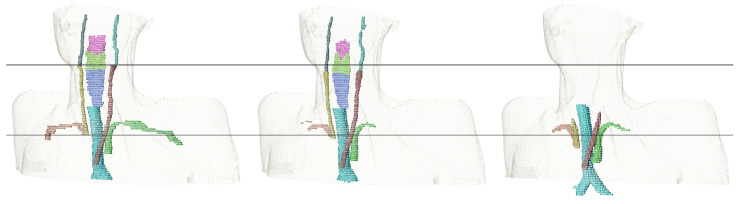
3D visualization of the subclavian artery (orange, green), the common carotid artery (yellow, brown), the internal carotid artery (dark green, cyan), the trachea (teal), and the constrictor muscles (pink, light green, blue). Contours are generated manually (**left**), by our trained nnU-Net models (**middle**), and by the TotalSegmentator (**right**). Horizontal black lines are there for heights comparison.

**Figure 6:**
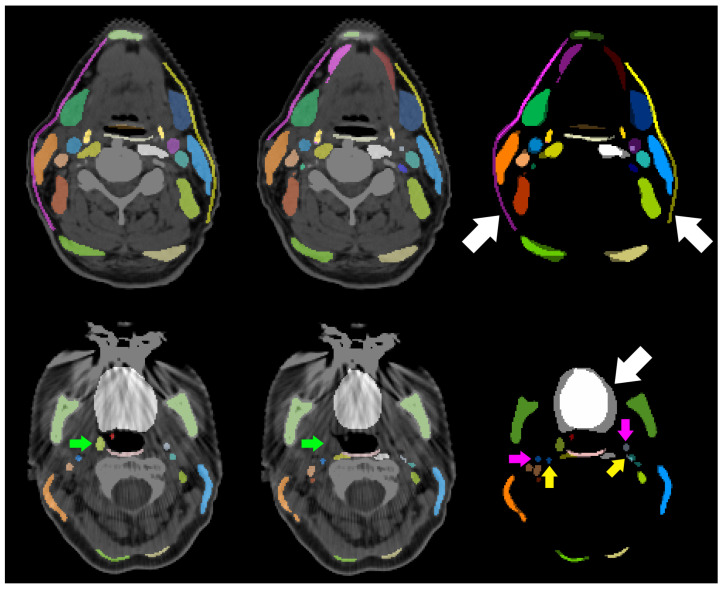
CT slices of two different patients with contours generated manually (**left**), contours generated by our trained nnU-Net models (**middle**), and the comparison of both contours without CT slice (**right**). White arrows indicate large deviations between both contours in the platysma (**top row**) and the tongue (**bottom row**). Deviations in the segmentations of the internal carotid artery are indicated by pink arrows (manual labels) and yellow arrows (predicted labels). The right tonsil (green arrow) is not visible.

The visual analysis of cases in which the *internal carotid artery (ICA)* shows especially low DICE and sDICE on both sides, results in four common reasons for deviations between the manual segmentation and its prediction: (a) the ICA is a thin structure, (b) the transition between internal carotid artery and common carotid artery varies, (c) the final slice, on which the ICA occurs cranially varies, and (d) due to metal in the mouth, CT artifacts occur in this area. Figure 5 shows the deviation between manual and predicted segmentation of the ICA due to inconsistent decision on the most cranial slice and the bottom row of Figure 6 shows metal artifacts.

For the *subclavian artery* similar reasons are resulting in small DICE_m_ and sDICE_m_: (a) the subclavian artery is a thin structure, (b) the transition between the right subclavian artery and the brachiocephalic artery varies, and (c) the lateral extension varies.

The visual analysis of the *superior constrictor muscles* and *middle constrictor muscles* also results in clear confusion at the area of transition between both structures, as well as the transition between the middle and the inferior constrictor muscles. This observation is supported by the above-median performance of their combination (i.e., constrictors (s., m., i.)). Training their combination, and differentiating the substructures in a rule-based post-processing, might be beneficial to the auto-segmentation of the constrictor muscles and similar cases.

The *digastric muscles* and the *posterior scalene muscles* show an (almost) below Q1 performance in DICE_m_ and sDICE_m_ with large standard deviations amongst test patients. DICE values range from [0–0.83] for the digastric muscles and [0–0.71 (0.81)] for the posterior scalene muscles. sDICE values deviate by more than 0.68 (digastric muscles) and 0.85 (posterior scalene muscles) between minimum and maximum. All predictions show greater accordance with the manual labels than the segmentations generated by the second observer (high inter-observer variability).

The *tongue* has an above-median DICE_m_, but a noticeable low sDICE_m_. Since the tongue is a theoretically easy to locate structure of above-average volume, the DICE_m_ does only marginally indicate problems with its segmentation. The sDICE_m_ signals inconsistencies in the precise outline of the tongue. Reasons are metal artifacts that occur predominantly in the area of the mouth which impair the precise segmentation of the tongue.

The right *platysma muscle* is an outlier in HD_m_. The analysis of individual cases shows a deviation of the manual labels in the frontal-dorsal direction and the cranial-caudal direction. Since the platysma muscle is a thin cutaneous muscle, it is sometimes barely visible in its most frontal and most dorsal extension. Thus, the network is trained on only a few extended examples. Auto-segmentations depict only the mostly visible inner extension of the platysma muscles.

### 4.2. Inter-Observer Variability, and Tracheostomy Analysis

**Figure 7:**
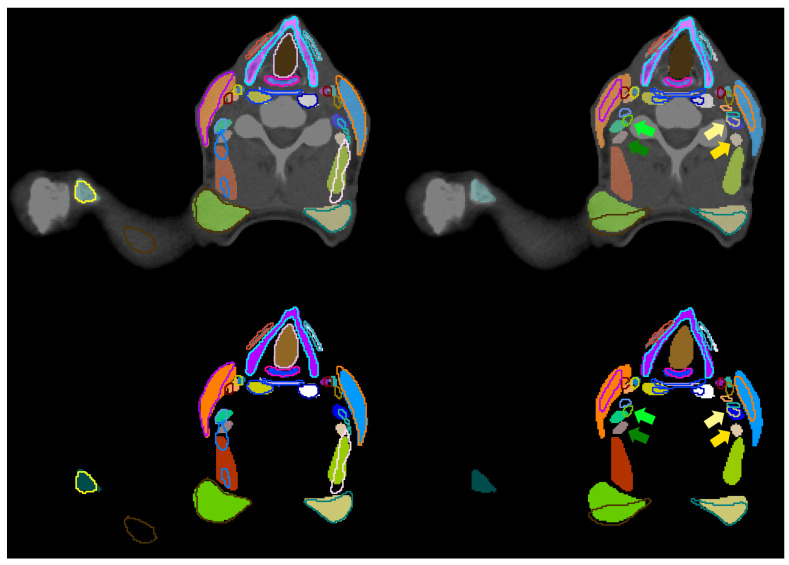
CT slice (top) with contours generated manually (area) for comparison (outline) with contours predicted by our trained nnU-Net models (**left**), and contours manually delineated by another trained observer (**right)**. The second set of contours does not contain all 71 structures (no outlines). Green (right) and yellow (left) arrows point to corresponding segmentations of the posterior scalene muscle generated by one observer (darker color) or the other (lighter color). The same contours whiteout CT slice are visualized in the bottom row.

The anatomical structures with an inter-observer variability outside the 3 σ interval around the mean in any of the three metrics or a value below the Q1 in DICE_m_ or sDICE_m_ or above the Q3 in HD_m_ were visually analyzed. Two systematic reasons are found that explain deviations. First, the lateral extension of the subclavian artery was inconsistent. Second, muscular structures were systematically segmented wider by one observer than by the other. This holds for the prevertebral muscles, the sternocleidomastoid muscles, the trapezius muscles and the digastric muscles. The deviation between all scalene muscles and the tonsils did not follow systematic reasons. Those structures are barely or not visible in the planning CT scans. Figure 6 shows this for the tonsil (green arrows). This results in largely deviating contours between both observers as visualized in the right column of Figure 7. No unambiguous reason can be given for the right internal carotid artery. As it is a thin structure that is difficult to segment, deviations occur in some central slices, while its left counterpart is much better aligned between both observers. No clear difference is visible between both sides of the patient CT scan.

Although the DL-models were trained on a distinct amount of patient data sets with tracheostomy, leaving out those patients from the analysis improves seventeen selected structures noticeably in almost all of the three metrics. Analyzing the deviation of the DICE_m_ and the sDICE_m_ for all other anatomical structures shows almost no change. Most of the 17 structures are in close proximity to the tracheostomy or the distortions in the larynx caused by tracheostomy.

### 4.3. Comparison to TotalSegmentator

Most anatomical structures that are automatically segmented by the TotalSegmentator framework (TS) are very similar to our own generated segmentations. For those structures that are deviating noticeable there is a common reason when analyzing the segmentations visually. Figure 5 includes the 3D comparison of those structures. The most common reason is the disagreement in the starting and ending position of elongated structures like the common carotid artery, the trachea, and the subclavian artery. Our manual segmentations for the common carotid arteries ends cranially at the artery’s bifurcation. Although caudally starting very similarly, the segmentations of the TS end approximately half way to the artery’s bifurcation, close to the cranial edge of the esophagus and the trachea. For the trachea, our manual labels exclude the bronchi, while the TS predicted segmentations include the right and left primary bronchi. Our manual labels for the subclavian artery exceed the TS generated labels laterally.

Deviations in the auto-segmentation of the thyroid gland result from patient-individual differences, rather than a systematic difference in the definition. Especially in patients that are equipped with a tracheostoma, the TS predictions deviate more from the manual segmentations than our own predictions. It might be, that in the training data set on which the TS model was trained, there were less or no patient data with a tracheostoma.

### 4.4. Impact on CTV Delineation

The delineation of CTVs should be targeted for auto-segmentation using DL algorithms. Following the international consensus guidelines of Grégoire et al. [27]. This study can be the basis for improved standardization and reduced workload. In the following section, the implications are analyzed that the prior described systematic deviations in the auto-segmentations of anatomical structures have on the clinical target volume delineation when following Grégoire et al. [27].

The predicted contour of the *internal carotid artery (ICA)* deviates caudally when transitioning into the common carotid artery (CCA) and its final slice cranially, as well as due to metal artifacts. Within the expert guidelines [27], the ICA is needed as the medial edge of Level II, the lateral edge of Level VIIa, and the medial edge of the Level VIIb. All these levels are transitioning into each other and the precise boundary becomes only relevant if some, but not all of these levels are irradiated. Since Level II begins caudally approximately where the CCA and ICA are transitioning, one might add the CCA as boundary into the rules when automating the delineation of Level II. The cranial edge of Level II is given by either the lateral process of C1 which the ICA always exceeds, or Level VIIb. The cranial edge of Level VIIb is the base of skull (jugular foramen) which the ICA reaches in all our test patients. Thus, the deviations introduced by the auto-segmentation of the ICA do not affect the CTVs’ delineation.

The predicted contour of the *subclavian artery (SuA)* deviates laterally and in its transition to the brachiocephalic artery. Within the expert guidelines [27], the SuA is needed as the posterior edge of the Level IVb. Caudally, this posterior boundary is cumulating both, the SuA and the brachiocephalic artery, such that their transition does not affect the delineation of the CTV. Also cranially, the lateral deviation of the SuA’s segmentation does not affect the posterior edge of the Level IVb. This is, because the SuA’s extension always exceeds the necessary boundary of Level IVb.

The predicted contour of the *inferior, middle and superior constrictor muscles (CM)* deviates caudally and cranially at the transitions between each other. Within the expert guidelines [27], the CM is needed as the anterior edge of Level VIIa which is bordering the superior or middle pharyngeal constrictor muscle. This boundary is cumulating both, the superior and middle CM, such that their transition does not affect the delineation of the CTV.

The predicted contour of the *platysma muscle (PM)* deviates in frontal and dorsal direction as well as in cranial and caudal direction. Within the expert guidelines [27], the PM is needed as caudal edge of Level Ia and Ib, lateral edge of Level Ib and Level V, and anterior edge of Level VIa. The caudal edge of Level Ia required sufficient delineations of the PM in its central regions which is shown consistently. The caudal edge of Level Ib is described by a plane independent of the PM. The PM only cuts this plane as it is the lateral border of Level Ib. For this, the central parts of the PM are relevant. Those are well-predicted. In the boundary descriptions of Level V and Level VIa, the skin is given as an alternative edge. Since the PM is a thin cutaneous muscle, the expert guidelines already account for its potential invisibility. In this case, there will be no further implications for the CTV delineation than the irradiation of the PM itself.

The predicted contour of the *anterior belly of the digastric muscle (aDM)* deviates unsystematically. Within the expert guidelines [27], the aDM is needed as caudal and lateral edge of Level Ia, and medial edge of Level Ib. For the caudal edge of Level Ia the aDM is not the primary boundary, but a substitute for the PM if the PM is not visible. Due to inconsistent delineations of the sDM, substituting the PM in this case might cause deviations in the caudal boundary of Level Ia. Nevertheless, as discussed before, the PM is often delineated well in the discussed region. Visually analyzing the data, as lateral edge of Level Ia, often the mandible is chosen. Further, as medial edge of Level Ib, often the Level Ia is chosen. Thus, the delineations we got from the clinics do not always spare the aDM. With our inconsistent delineations, we cannot improve this situation and spare the aDM reliably. No solution can be provided for cases in which Level Ib is irradiated while Level Ia is not.

The predicted contour of the *posterior scalene muscle (pSM)* deviates unsystematically. Within the expert guidelines [27], the scalene muscles are needed as medial edge of Level II, Level III, Level IVa, Level V, Level Vc, posterior edge of Level IVa, and lateral edge of Level IVb. Although not specified precisely, the visual analysis shows that most boundaries are given by the anterior scalene muscle. The pSM potentially plays a role in delineating the medial edge of Level V caudally. Here, the confusion between different scalene muscles does not affect CTV delineation, but the pSM could be unintentionally irradiated if contoured erroneously.

The predicted contour of the *tongue* and the *tonsils* deviate unsystematically due to metal artefacts and missing soft tissue contrast. Since both structures are not used as a boundary definition, but only as selection criterion for nodal levels in the expert guidelines [27], the CTV delineation is not affected by distortions of these two structures.

### 4.5. Limitations and Future Research Directions

In our study, we segmented 71 anatomical structures. With additional tools like the TotalSegmentator, the set of structures can be further extended. Nevertheless, even including multiple models, there are still anatomical structures that are segmented neither previously nor in this study. Thus, the dense segmentation of all anatomical structures in the human body is still an issue. Future research should focus on bringing different segmentation models together to generate data sets with dense labels so that the observed positive effects of dense annotations can be exploited.

For this, the large inter-observer variability indicates upcoming problems related to this topic. In our opinion, better agreement of structures’ definitions should be reached, before dense annotations can be generated expediently. Their precise delineation could be supported by additional multi-modal images. We suggest to use MRI scans which have better soft tissue contrast in addition CT scans for the segmentation of soft tissue structures.

Not all necessary structures are covered for the auto-segmentation of all CTV levels in the head and neck area. Structures like the posterior belly of the digastric muscle, the mylo-hyoid muscle, the transversal cervical vessel and the infrahyoid (strap) muscles are missing for completeness. Further, some segmented structures do not lead to sufficient prediction accuracy to be spared (e.g., the anterior belly of the digastric muscles). Completing the prerequisites for generating a guideline conform CTV automatically, additional manual labels need to be generated on which new models can be trained for their auto-segmentation. Improvements for the anterior belly of the digastric muscles and the platysma muscle are expected from the use of additional MRI scans.

Although our training data set was very diverse, the number of training and test samples was too low to train the models to identify each image feature and each patient condition. Thus, patients with tracheostomy led to worse segmentation accuracies. The same might hold for postoperative patients, different stages of contrast agents, or different resolutions of CT scans. Additional data sets might improve the results on underrepresented image features.

In the future, we aim to construct guideline conform CTV delineations by extracting the necessary anatomical boundaries from the generate labels of the presented 71 anatomical structures. These boundaries can be combined following the expert guidelines to form all of the ten levels in the head and neck area which are selected for radiation therapy dependent on the location of the primary tumor. All discussed segmented anatomical structures show sufficient accuracy for this method of CTV generation. Thus, the automatization of CTV delineation becomes independent of inconsistent training and test labels, while providing the desired standardization and becoming more easy to adapt to changes in the guidelines than common segmentation methods.

## 5. Conclusions

In this study, we have automatically segmented 71 anatomical structures in the head and neck area relevant for CTV delineation. Most of these structures have not been automatically segmented before. We analyzed systematic deviations of anatomical structures that showed mean DICE, mean HD or mean sDICE values below the Q1 (above the Q3, respectively) and their impact on the automation of CTV delineation. No deviation is expected to be inferior to the current clinical practice.

These results are a step forward towards dense annotations and the auto-segmentation of guideline conform CTV delineation.

## Figures and Tables

**Table 2:** List of all segmented anatomical structures (right (r), left (l)) and their combinations (e.g., *sternum (M., C.)*) sorted by tissue type. For each structure, the HD (95) and sDICE (2 mm) (mean ± standard deviation) between the manual contours and our models’ predicted contours (pred.) is given, as well as the inter-observer variability in HD (95) and sDICE (2 mm) (calculation based on a single patient data set). Asterisks (^*^) indicates inter-observer variability values outside the 3 σ interval given by the mean and standard deviation of the models’ comparison to the manual labels.

		HD (95)		sDICE (2 mm)	
	Structure	Pred. vs. Manual	Inter-Observer	Pred. vs. Manual	Inter-Observer
Air	Auditory Canal (l)	5.16 ± 2.94		0.88 ± 0.08	
	Auditory Canal (r)	4.76 ± 3.16		0.89 ± 0.09	
	Larynx (air)	6.74 ± 4.13		0.89 ± 0.06	
	Lung (l)	1.42 ± 1.00		0.97 ± 0.03	
	Lung (r)	1.50 ± 0.86		0.98 ± 0.02	
	Trachea	6.87 ± 5.49		0.90 ± 0.08	
Bones	Cheek Bone (l)	4.23 ± 2.89		0.92 ± 0.05	
	Cheek Bone (r)	4.36 ± 3.37		0.92 ± 0.07	
	Clavicle (l)	1.33 ± 0.67		0.98 ± 0.02	
	Clavicle (r)	1.25 ± 0.49		0.98 ± 0.01	
	Hyoid Bone	3.23 ± 3.77	1.96	0.95 ± 0.06	0.97
	Mandible	2.31 ± 1.67	2.77	0.96 ± 0.04	0.88
	*Sternum (M., C.)*	1.98 ± 1.63		0.97 ± 0.04	
	Sternum Corpus	5.87 ± 6.69		0.87 ± 0.20	
	Sternum Manubrium	3.99 ± 4.18	3.00	0.93 ± 0.08	0.93
	Styloid Process (l)	5.72 ± 9.58		0.92 ± 0.13	
	Styloid Process (r)	2.01 ± 0.97		0.97 ± 0.03	
	Vertebra C1	3.07 ± 1.24	3.16	0.93 ± 0.04	0.90
Ca.	Cricoid Cartilage	6.15 ± 3.30	3.16	0.82 ± 0.14	0.92
	Thyroid Cartilage	2.40 ± 2.10	0.98	0.96 ± 0.04	0.98
Gland	Submandibular Gland (l)	5.04 ± 4.28		0.85 ± 0.15	
	Submandibular Gland (r)	4.50 ± 2.69		0.80 ± 0.23	
	Thyroid Gland	6.12 ± 9.45		0.89 ± 0.13	
	Brachiocephalic Artery	3.90 ± 2.66	3.00	0.89 ± 0.09	0.96
	Brachiocephalic Vein (l)	3.53 ± 1.58	6.00	0.90 ± 0.08	0.88
	Brachiocephalic Vein (r)	4.88 ± 2.09	4.08	0.86 ± 0.07	0.85
	Common Carotid Artery (l)	5.01 ± 7.04	2.94	0.94 ± 0.06	0.94
	Common Carotid Artery (r)	3.48 ± 2.69	4.38	0.92 ± 0.07	0.81
Vessels	Internal Carotid Artery (l)	7.53 ± 8.95	11.17	0.84 ± 0.12	0.38 ^*^
	Internal Carotid Artery (r)	13.85 ± 15.86	4.38	0.75 ± 0.20	0.80
	Internal Jugular Vein (l)	9.57 ± 23.20	9.00	0.91 ± 0.10	0.64
	Internal Jugular Vein (r)	8.25 ± 14.72	6.20	0.87 ± 0.14	0.73
	Subclavian Artery (l)	16.36 ± 19.40	81.22 ^*^	0.84 ± 0.11	0.54
	Subclavian Artery (r)	10.27 ± 12.35	75.01 ^*^	0.83 ± 0.12	0.42 ^*^
	*Constrictors (s., m., i.)*	7.19 ± 6.40	3.00	0.89 ± 0.08	0.95
	Inferior Constrictor	7.10 ± 6.16	2.77	0.82 ± 0.16	0.95
	Middle Constrictor	9.66 ± 6.41	9.00	0.72 ± 0.18	0.88
	Superior Constrictor	11.23 ± 8.38	9.00	0.73 ± 0.22	0.75
	Digastric (l)	6.08 ± 3.90	6.30	0.73 ± 0.22	0.58
	Digastric (r)	8.52 ± 5.28	6.96	0.64 ± 0.30	0.52
	Levator Scapulae (l)	3.86 ± 2.05		0.92 ± 0.05	
	Levator Scapulae (r)	5.26 ± 2.87		0.88 ± 0.07	
	Platysma (l)	13.02 ± 9.59		0.82 ± 0.12	
	Platysma (r)	19.40 ± 11.75		0.75 ± 0.17	
	Prevertebral (l)	7.35 ± 8.25	6.86	0.90 ± 0.05	0.75
	Prevertebral (r)	7.29 ± 8.51	6.28	0.91 ± 0.05	0.73 ^*^
	*Scalene (an., me., p.) (l)*	5.74 ± 3.20	13.09	0.86 ± 0.08	0.64
	*Scalene (an., me., p.) (r)*	7.59 ± 5.19	15.80	0.82 ± 0.10	0.21 ^*^
Muscles	Anterior Scalene (l)	7.36 ± 9.67	15.00	0.92 ± 0.07	0.85
	Anterior Scalene (r)	8.19 ± 9.73	16.69	0.89 ± 0.07	0.17 ^*^
	Medius Scalene (l)	6.06 ± 2.84	9.82	0.81 ± 0.10	0.42 ^*^
	Medius Scalene (r)	7.63 ± 4.11	19.16	0.78 ± 0.11	0.21 ^*^
	Posterior Scalene (l)	14.84 ± 8.84	17.71	0.56 ± 0.23	0.14
	Posterior Scalene (r)	17.16 ± 16.53	19.45	0.57 ± 0.30	0.10
	Sternothyroid (l)	4.48 ± 2.36		0.89 ± 0.08	
	Sternothyroid (r)	4.87 ± 2.03		0.89 ± 0.08	
	Sternocleidomastoid (l)	4.94 ± 5.34	22.57 ^*^	0.92 ± 0.08	0.50 ^*^
	Sternocleidomastoid (r)	12.31 ± 24.65	20.98	0.88 ± 0.15	0.54
	Thyrohyoid (l)	4.16 ± 2.68	3.10	0.86 ± 0.12	0.91
	Thyrohyoid (r)	3.08 ± 1.18	4.04	0.90 ± 0.07	0.87
	Trapezius (l)	2.38 ± 0.76	12.96 ^*^	0.96 ± 0.03	0.69 ^*^
	Trapezius (r)	2.43 ± 0.59	9.42 ^*^	0.95 ± 0.04	0.71 ^*^
	Tongue	13.29 ± 5.51		0.43 ± 0.17	
	Esophagus	6.15 ± 5.92		0.88 ± 0.10	
	Hard Palate	7.60 ± 4.08		0.73 ± 0.12	
	Hypopharynx	6.74 ± 3.85	2.94	0.83 ± 0.12	0.93
	Nasal Cavity (l)	2.30 ± 0.79		0.96 ± 0.02	
	Nasal Cavity (r)	2.26 ± 0.74		0.96 ± 0.02	
	Nasopharynx	4.84 ± 3.35	4.94	0.84 ± 0.12	0.72
	Oral Cavity	7.56 ± 3.80		0.67 ± 0.12	
	Oropharynx	6.40 ± 4.89	6.00	0.88 ± 0.09	0.83
	*Pharynx (nasop., orop., hyp.)*	5.15 ± 2.78	3.30	0.89 ± 0.06	0.91
	Skin	1.88 ± 1.08		0.96 ± 0.05	
	Soft Palate	9.33 ± 7.89		0.75 ± 0.18	
	Tonsil (l)	10.57 ± 8.90	15.00	0.20 ± 0.23	0.26
	Tonsil (r)	11.15 ± 8.19	15.13	0.28 ± 0.27	0.31

**Table 3:** Mean DICE, mean HD (95) and mean sDICE (2 mm) for all test patients without tracheostomy (#15). Seventeen structures are selected for that the mean DICE and mean sDICE (2 mm) increased the most when compared to the values resulting from the analysis including all patients. The deviation between the analysis including all patients and the analysis excluding patients with tracheostomy is given in brackets.

Structure	DICE	HD (95)	sDICE (2 mm)
Trachea	0.92 (0.13)	5.64 (− 7.40)	0.93 (0.16)
Hyoid Bone	0.83 (0.12)	2.31 (−7.32)	0.96 (0.09)
Thyroid Gland	0.84 (0.14)	5.90 (−1.32)	0.92 (0.18)
Internal Carotid Artery (r)	0.57 (0.10)	11.77 (−12.50)	0.77 (0.10)
Internal Jugular Vein (r)	0.78 (0.15)	8.09 (−0.98)	0.89 (0.13)
Constrictors (s., m., i.)	0.59 (0.19)	7.14 (−0.32)	0.90 (0.10)
Middle Constrictor	0.48 (0.21)	9.17 (−2.93)	0.75 (0.15)
Superior Constrictor	0.52 (0.23)	11.32 (0.50)	0.75 (0.14)
Digastric (r)	0.51 (0.30)	7.56 (−5.75)	0.69 (0.33)
Platysma (r)	0.54 (0.18)	17.61 (−15.24)	0.78 (0.20)
Sternothyroid (r)	0.60 (0.21)	4.66 (−3.01)	0.91 (0.28)
Sternocleidomastoid (l)	0.86 (0.12)	3.63 (−7.86)	0.93 (0.09)
Sternocleidomastoid (r)	0.85 (0.26)	5.17 (−42.80)	0.92 (0.26)
Thyrohyoid (r)	0.57 (0.09)	2.85 (−1.79)	0.91 (0.12)
Esophagus	0.82 (0.12)	5.41 (−4.44)	0.90 (0.11)
Hypopharynx	0.68 (0.23)	5.95 (−4.73)	0.86 (0.18)
Soft Palate	0.63 (0.16)	8.64 (−4.12)	0.78 (0.14)

**Table 4:** Subset of segmented anatomical structures of this study for which segmentation labels are also available in the TotalSegmentator toolkit [11]. For each structure, the DICE (mean ± standard deviation) between the TS predicted contour (pred.) and the manual contour is given, as well as the decline in mean DICE (diff.) between the TS predicated contour and our models’ predicted contour.

Structure	Pred. vs. Manual	Diff.
Lung (l)	0.98 ± 0.01	−0.01
Lung (r)	0.98 ± 0.01	−0.01
Trachea	0.80 ± 0.06	−0.10
Clavicle (l)	0.89 ± 0.03	−0.04
Clavicle (r)	0.88 ± 0.02	−0.06
*Sternum (M., C.)*	0.90 ± 0.02	−0.02
Vertebra C1	0.81 ± 0.04	−0.05
Thyroid Gland	0.71 ± 0.14	−0.10
Brachiocephalic Artery	0.75 ± 0.07	−0.09
Brachiocephalic Vein (l)	0.76 ± 0.10	−0.05
Brachiocephalic Vein (r)	0.72 ± 0.08	−0.10
Common Carotid Artery (l)	0.64 ± 0.13	−0.17
Common Carotid Artery (r)	0.55 ± 0.18	−0.23
Subclavian Artery (l)	0.67 ± 0.10	−0.07
Subclavian Artery (r)	0.65 ± 0.14	−0.09
Esophagus	0.77 ± 0.09	−0.04

**Table 5:** Subset of segmented anatomical structures of this study for which segmentation labels are also available in the TotalSegmentator toolkit [11]. For each structure, the HD and the sDICE (mean ± standard deviation, each) between the TS predicted contour (pred.) and the manual contour is given, as well as the decline in mean HD and sDICE (diff.) between the TS predicated contour and our models’ predicted contour.

	HD (95)		sDICE (2 mm)	
Structure	Pred. vs. Manual	Diff.	Pred. vs. Manual	Diff.
Lung (l)	2.18 ± 1.31	0.76	0.97 ± 0.03	−0.01
Lung (r)	1.91 ± 1.31	0.41	0.97 ± 0.01	0.00
Trachea	16.04 ± 6.73	9.17	0.80 ± 0.09	−0.10
Clavicle (l)	2.54 ± 1.82	1.21	0.96 ± 0.03	−0.02
Clavicle (r)	2.83 ± 1.69	1.57	0.94 ± 0.03	−0.04
*Sternum (M., C.)*	2.98 ± 1.45	1.00	0.94 ± 0.03	−0.03
Vertebra C1	3.70 ± 1.52	0.63	0.90 ± 0.06	−0.03
Thyroid Gland	8.89 ± 8.70	2.77	0.79 ± 0.15	−0.11
Brachiocephalic Artery	9.29 ± 5.16	5.39	0.80 ± 0.08	−0.09
Brachiocephalic Vein (l)	5.82 ± 2.07	2.28	0.86 ± 0.08	−0.04
Brachiocephalic Vein (r)	7.68 ± 2.96	2.80	0.79 ± 0.08	−0.07
Common Carotid Artery (l)	25.15 ± 17.16	20.14	0.80 ± 0.13	−0.13
Common Carotid Artery (r)	28.41 ± 20.01	24.94	0.71 ± 0.17	−0.22
Subclavian Artery (l)	23.94 ± 16.66	7.58	0.79 ± 0.10	−0.05
Subclavian Artery (r)	20.88 ± 17.13	10.61	0.75 ± 0.14	−0.08
Esophagus	9.80 ± 9.62	3.65	0.85 ± 0.10	−0.03

## Data Availability

Data sets used in this study were taken either from the Cancer Imaging Archive or in-house studies with patients from UKHD. Restrictions apply to the availability of the TCIA data sets. Data are available at https://wiki.cancerimagingarchive.net/pages/viewpage.action?pageId=39879146#3987914647deb804b80d40149cf58b123547480d (accessed on 6 April 2022) with the permission of TCIA. In-house pseudonymized data are available on request from the corresponding author for research purposes if in line with ethics committee requirements. The data are not publicly available due to missing explicit patient consent for data publication in this retrospective study.

## Abbreviations

The following abbreviations are used in this manuscript:

aDM	Anterior Belly of the Digastric Muscle
CCA	Common Carotid Artery
CM	Constrictor Muscle
CT	Computertomographie
CTV	Clinical Target Volume
DICE	Sørensen–Dice Coefficient
DL	Deep Learning
HD	Hausdorff Distance
ICA	Internal Carotid Artery
OAR	Organs At Risk
PM	Platysma Muscle
pSM	Posterior Scalene Muscle
Q1	25th Percentile
Q3	75th Percentile
sDICE	Surface DICE
SuA	Subclavian Artery
TS	TotalSegmentator

## Appendix A

### Appendix A.1. Standard Operation Procedure

1. Nasopharynx
  - Cranial boundary: up to the nasal septum
  - Caudal boundary: from the hard palate
2. Oropharynx
  - Cranial boundary: from the hard palate
  - Caudal boundary: epiglottis
3. Hypopharynx
  - Cranial boundary: epiglottis
  - Caudal boundary: transition to the esophagus, along with the caudal end of the cricoid cartilage
  - Segmentation note: No clear caudal boundary, orientation is based on the larynx-air structure
4. Tongue (muscle)
  - Bounded by the oral cavity
  - Caudal boundary: tongue base (ambiguous border)
5. Thyroid cartilage
  - Segment in the larynx window
  - Boundary: entire cartilage structure (typical shape was always recognizable in 3D view)
6. Sternocleidomastoid muscle
  - Cranial boundary: mastoid cells, up to the skull
  - Caudal boundary: clavicle and sternal manubrium, occasional branching near the origin may be visible
7. Thyroid gland
  - Bright structure, merging caudally, variable cranial boundary
8. Hyoid bone
  - Segment in the bone window
  - Boundary: entire bone structure (typical shape was always recognizable in 3D view)
9. Cricoid cartilage
  - Segment in the larynx window
  - Boundary: entire cartilaginous structure (typical shape usually visible in 3D view)
  - Special note: Caudal boundary simultaneously limits hypopharynx, larynx air, and inferior constrictor
10. Pharyngeal constrictor muscles (superior, medius, inferior)
  - S. from the level of upper jaw teeth caudally
  - M. from hyoid cranially (both structures "meet" in the middle)
  - I. from hyoid caudally to the caudal end of the cricoid cartilage
11. Esophagus
  - Cranial boundary: caudal end of the cricoid cartilage
  - Caudal boundary irrelevant for head-neck region, as the esophagus ends in the stomach
12. C1/vertebral bodies
  - Segment in the bone window
13. Soft palate
  - Cranial boundary: transition to hard palate
  - Caudal boundary: uvula
14. Hard palate
  - Segment in the larynx window
15. Larynx
  - Cranial boundary: epiglottis
  - Caudal boundary: caudal end of the cricoid cartilage
16. Mandible
  - Segment in the bone window
  - Teeth not segmented
17. Digastric muscle
  - Cranial boundary: mandible
  - Caudal boundary: until no longer visible
18. Nasal cavities
  - Cranial boundary: until no longer visible
  - Caudal boundary: together with nasopharynx
  - Note: Exclude ethmoid cells
19. Oral cavities
  - Includes tongue, uvula
20. External Auditory Canal
21. Tonsils
  - Bilateral at the level of uvula
22. Common Carotid Artery
  - Cranial boundary: until artery bifurcation
  - Caudal boundary: branching from brachiocephalic trunk
23. Sternal manubrium
  - Segment in the bone window
  - Note: Manubrium is posterior at transition with corpus sterni
24. Sternum body
  - Segment in the bone window
  - Note: Corpus is anterior at transition with manubrium
25. Clavicle
  - Segment in the bone window
26. Zygomatic arch
  - Segment in the bone window
  - Ventral boundary: continuation from posterior edge of maxillary sinus
  - Dorsal boundary: up to mastoid cells
27. Styloid process
  - Segment in the bone window
  - Cranial boundary: first slice where not connected to mastoid
  - Caudal boundary: until no longer visible
28. Lung
  - Segment in the lung window
  - Often already exists
  - ‘Region growing’ with upper threshold = −300 and ‘remove holes’, but avoid including trachea/air outside the patient (sometimes segmented, correct manually)
29. Trachea
  - Cranial boundary: larynx air
  - Caudal boundary: bifurcation
  - Excludes bronchi
30. Internal Carotid Artery
  - Cranial boundary: entry into the skull
  - Caudal boundary: separation of common carotid
31. Internal Jugular Vein
  - Cranial boundary: entry into the skull
  - Caudal boundary: brachiocephalic vein
32. Trapezius muscles
  - Cranial boundary: skull
  - Caudal boundary: from the spine
  - Note: At the clavicle, trapezius also extends anteriorly, creating a tight “hole” in segmentation where tendon lies
33. Platysma Muscle
  - Boundaries not clear but segmented as long as visible course toward mandible
34. Brachiocephalic Artery
  - Cranial boundary: up to division into common carotids
  - Caudal boundary: from aortic arch
35. Brachiocephalic vein
  - Cranial boundary: up to division into IJV
  - Caudal boundary: from SVC division
36. Submandibular Gland
  - Segment as long as visible within platysma
37. Levator Scapulae Muscle
  - Cranial boundary: as far as possible
  - Caudal boundary: from scapula
38. Scalenus muscles (anterior, medius, posterior)
  - A. and M. around subclavian artery
  - A. and M. originate from first rib
  - P. often unclear, originates from second rib
  - All three structures traced cranially as far as possible
39. Subclavian Artery
  - Lateral boundary: up to cranial boundary of sternum
40. Skin
  - Adopt from outline or external contour and correct significant errors from automatic contouring
41. Sterno-thyroid muscle
  - Cranial boundary: first slice where thyroid cartilage is ventrally united
  - Caudal boundary: first slice from manubrium
42. Thyro-hyoid muscle
  - Cranial boundary: first slice where hyoid is visible
  - Caudal boundary: first slice after sternothyroid
43. Pre-vertebral muscles (longus colli + longus capiti)
  - Cranial boundary: up to visible dens axis
  - Caudal boundary: as far as possible

### Appendix A.2. Previously Reported DICE Values for Comparison

**Table A1:** Previously reported DICE values (mean ± standard deviation) between contours predicted by different deep learning methods and manual labels.

Structure	Previously Reported DICE (Mean ± Std.)
Mandible	0.86 ± 0.12 ^1^ [56], 0.90 ± 0.04 [54], 0.91 ± 0.02 [55], 0.94 ± 0.02 [57], 0.94 ± 0.01 [52], 0.99 ± 0.01 [55]
Submandibular Gland (r)	0.73 ± 0.09 [54], 0.78 ± 0.10 [52], 0.79 [51], 0.95 ± 0.07 [55], 0.98 ± 0.03 [55]
Submandibular Gland (l)	0.70 ± 0.13 [54], 0.77 ± 0.12 [52], 0.79 [51], 0.91 ± 0.08 [55], 0.97 ± 0.05 [55]
Thyroid Gland	0.83 ± 0.08 [52], 0.90 ± 0.02 [57]
Internal Carotid Artery (r)	0.81 [49], 0.86 ± 0.02 [50]
Internal Carotid Artery (l)	0.81 [49], 0.86 ± 0.02 [50]
Superior Constrictor	0.67 ± 0.11 [60], 0.76 ± 0.13 [55], 0.83 ± 0.15 [55]
Middle Constrictor	0.60 ± 0.19 [60], 0.76 ± 0.10 [55], 0.84 ± 0.01 [55]
Inferior Constrictor	0.65 ± 0.12 [60], 0.71 ± 0.21 [55], 0.80 ± 0.24 [55]
*Constrictors (s., m., i.)*	0.52 [51], 0.64 ± 0.13 [57], 0.68 ± 0.08 [52]
Esophagus	0.85 ± 0.10 [55], 0.91 ± 0.03 [52], 0.93 ± 0.07 [55]
Oral Cavity	0.85 ± 0.10 [55], 0.90 ± 0.04 [57], 0.91 ± 0.03 [52], 0.93 ± 0.07 [55]

^1^ The values are only estimated from presented graphs in the referenced paper.
